# Probiotic supplementation as a nutritional strategy for the prevention and management of sarcopenia in older adults

**DOI:** 10.3389/fcimb.2026.1801885

**Published:** 2026-08-13

**Authors:** De’en Wang, Wei Yang, Xiaofang Qin

**Affiliations:** 1 Department of Geriatrics, The Affiliated Huaian No.1 People’s Hospital of Nanjing Medical University, Huaian, China; 2 Department of Ultrasound Medicine, The Affiliated Huaian No.1 People’s Hospital of Nanjing Medical University, Huaian, China

**Keywords:** gut–muscle axis, healthy aging, precision nutrition, probiotics, sarcopenia

## Abstract

**Introduction:**

Sarcopenia, defined as the loss of skeletal muscle mass, strength, and functional capacity over time, poses a significant health burden among older adults. A growing body of evidence indicates that the gut microbiota contributes to inflammation and metabolic regulation, mitochondrial function, and anabolic signaling, thereby shaping the gut–muscle axis. Probiotic supplementation has gained traction as a nutritional approach for the regulation of microbiome-derived metabolites. The present study aims to assess the rationale for probiotic supplementation to prevent and manage sarcopenia in older individuals.

**Methods:**

Using data from NHANES (2011–2018; n = 3,500), UK Biobank (n = 5,000), and GMrepo (n = 800), we examined associations between probiotic exposure and muscle outcomes, inflammation, and microbiome diversity. C2C12 myotubes were used in vitro under probiotic-conditioned media (PCM). The interactions of short-chain fatty acids (SCFAs) with the regulatory proteins (mTOR, AMPK, NF-κB) were evaluated using molecular docking simulations. Random Forest and XGBoost models were used to predict sarcopenia risk using integrated multimodal features, with a 70% training and 30% validation split.

**Results:**

Probiotic users exhibited greater handgrip strength (UK Biobank: 27.2 ± 4.8 kg vs. 25.1 ± 5.2 kg), higher fat-free mass (57.4 ± 10.6 kg vs. 56.2 ± 11.0 kg), and lower inflammatory burden compared to non-users. PCM increased fusion index (85.6 ± 2.3% vs. 78.9 ± 3.2%, p < 0.01), myotube diameter (45.2 ± 6.3 μm vs. 40.1 ± 5.7 μm, p < 0.05), and MyoD (2.1-fold), Myogenin (1.8-fold), and MyHC (2.2-fold). NF-κB and TNF-α were decreased (~0.60-fold and 0.65-fold, respectively), and PGC-1α was increased (1.9-fold). Butyrate docking revealed high binding affinity for AMPK (−6.9 kcal/mol), mTOR (−6.4 kcal/mol), and NF-κB (−6.0 kcal/mol). The performance metrics achieved highly in XGBoost with 88% accuracy.

**Discussion:**

This comprehensive framework positions probiotics as a precision nutrition intervention for sarcopenia prevention, targeting muscle-sparing effects through microbiome modulation that stimulates coordinated immunometabolic and anabolic actions, with AI-enhanced, individualized risk prediction for healthy aging.

## 1 Introduction

Sarcopenia, the loss of muscle quantity and quality in aging, has emerged as a major worldwide public health threat and is defined as the progressive and generalized loss of skeletal muscle mass, strength, and functional performance with age (Ji et al., 2022). In older adults, sarcopenia is strongly associated with frailty, disabilities, and metabolic dysfunction, as well as increased falls, increased hospitalizations, reduced quality of life, and mortality (Nishikawa et al., 2021). Given the increasing rate of worldwide older population, sarcopenia poses a clinical challenge and a major socioeconomic drain on health systems (Grosman and Kalichman, 2025). Historically, sarcopenia has been considered primarily as an age-related phenomenon of neuromuscular degeneration, hormonal deficits, decreasing levels of physical activity, and insufficient nutrient intake (Gustafsson and Ulfhake, 2021). Recent research has increasingly acknowledged sarcopenia as a systemic, multifactorial condition mediated by direct and indirect interactions between metabolic, inflammatory, immunological, and environmental factors (Ramos-Lopez et al., 2021).

Chronic low-grade inflammation, mitochondrial dysfunction, dysregulation of protein synthesis, anabolic resistance, and metabolic dysregulation are increasingly recognized as key biological drivers of muscle loss with aging (García-Domínguez, 2025). High levels of inflammatory mediators, including C-reactive protein (CRP) and TNF-α, as well as NF-κB signaling mediators, contribute to muscle protein degradation, mitochondrial dysfunction, and decreased regenerative capacity (Stanimirovic et al., 2022). Concurrently, decreased mitochondrial biogenesis, dysfunctional energy metabolism, and reduced activation of anabolic pathways, such as mTOR signaling, prevent optimal muscle preservation and reparative function (Chen et al., 2023). These interrelated processes indicate sarcopenia is not simply a local muscular disease but a systemic immunometabolic disease of aging needing integrated prevention and treatment rather than discrete interventions (Yu et al., 2024).

The gut microbiota has recently attracted attention as an important modulator of host metabolism, immune homeostasis, inflammation, and energy balance, thereby giving rise to the concept of the gut–muscle axis (Liu et al., 2022). Thus, the gut microbiome affects skeletal muscle physiology through multiple mechanisms, including modulation of systemic inflammation, regulation of nutrient absorption, production of bioactive metabolites, and modulation of host metabolic signaling pathways (Ma et al., 2025). Microbial fermentation of dietary fibers generates short-chain fatty acids (SCFAs) such as acetate, propionate, and butyrate, which regulate mitochondrial biogenesis, inflammatory signaling, insulin sensitivity, and anabolic metabolism (Rios-Morales et al., 2022). These metabolites serve as both fuel sources and signaling molecules that modulate key regulatory processes associated with muscle metabolism and inflammation (Baker and Rutter, 2023).

Probiotics, live organisms with health benefits given in a sufficient dose, are a practical and scalable solution to change gut microbiota composition and action (Britton et al., 2021). Recent studies also indicate that probiotics may decrease systemic inflammation, normalize metabolism, support mitochondrial function and promote muscle homeostasis (Turimov Mustapoevich and Kim, 2023). Preclinical studies have shown that specific metabolites produced by probiotics can induce myogenic differentiation, demonstrate anti-inflammatory signal responses, and enhance muscle regenerative capacity (Herich et al., 2025). Then, observational studies performed on aging populations also indicated associations between probiotic consumption and better physical performance and lower risk of frailty, respectively. Nevertheless, while the interest in probiotics is increasing, the mechanistic pathways linking them to muscle sparing are still incompletely understood, and the clinical translation, limited by fragmented evidence across various disciplines (Giron et al., 2022; Huang et al., 2024).

Recent advances in multimodal artificial intelligence have shown promise in clinically meaningful prediction tasks that integrate diverse data types to characterize disease phenotypes and stratify risk. For example, multimodal *digital biopsy* approaches combining radiologic imaging, genomic profiles, and clinical data have demonstrated improved non-invasive phenotyping and risk assessment in clinical cohorts (Chen et al., 2026; Ding et al., 2025a). Similarly, frameworks that fuse radiomic features, clinical variables, and biological markers have been successfully applied to recurrence prediction and prognostic modeling, highlighting the utility of multimodal fusion in translational AI (Ding et al., 2025c; Ding et al., 2025b; Ding et al., 2026). These developments underscore the potential of multimodal analytic strategies to enhance predictive performance and clinical interpretability beyond traditional single-modality models.

**Figure 1:**
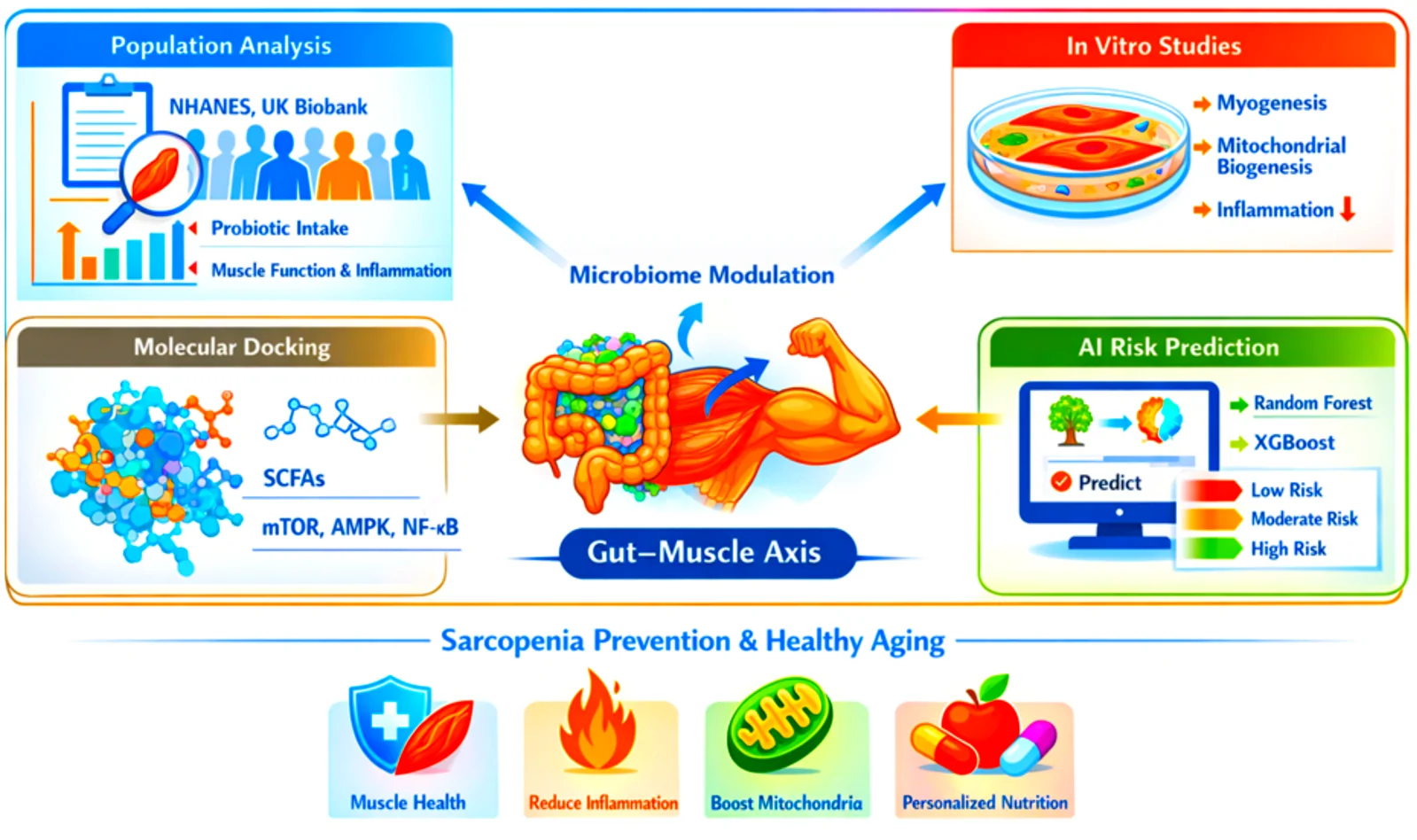
Probiotics and gut-muscle axis in sarcopenia prevention.

Hence, this study aims to fill this gap by developing a multi-scale, integrative framework to examine probiotic supplementation as a nutritional strategy against sarcopenia in older adults across the life span. This work aims to integrate–(i) large-scale human gut and skeletal muscle dataset analysis, (ii) *in vitro* mechanistic validation using skeletal muscle cell model, (iii) in silico molecular docking of microbiome-derived metabolites–with essential gut interactions or effector (regulatory) proteins, and (iv) machine learning–based predictive modeling; to arrive at a systems-level understanding of the gut–muscle axis. **Figure 1** illustrates a comprehensive approach to studying gut-muscle health and sarcopenia prevention. It integrates population analysis (NHANES, UK Biobank) to explore the relationship between probiotic intake and muscle function, microbiome modulation to investigate how probiotics affect muscle health, and *in vitro* studies focusing on myogenesis, mitochondrial biogenesis, and inflammation. Molecular docking of SCFAs with proteins like mTOR, AMPK, and NF-κB provides mechanistic insights, while AI-driven prediction models (Random Forest, XGBoost) help assess sarcopenia risk. The ultimate goal is to improve muscle health, reduce inflammation, and support healthy aging through personalized nutrition and interventions.

## 2 Materials and methods

### 2.1 Overall study design

Here, we utilized a three-level integrated research design, including secondary analysis of human population datasets, *in vitro* skeletal muscle experiments, and in silico computational validation to assess whether probiotic supplementation could serve as a nutritional strategy to prevent and combat sarcopenia in older adults. The human element employed de-identified, publicly available datasets to characterize probiotic exposure and sarcopenia-related outcomes, while *in vitro* experiments were performed to determine concordant biological mechanisms at the cellular level. The results were further investigated using in silico analyses to describe molecular interactions and to develop predictive models of the relationships among probiotic exposure, gut microbiota, inflammation, and muscle.

### 2.2 National health and nutrition examination survey

Data were obtained from NHANES cycles during 2011–2018 and were limited to participants aged ≥60 years with complete dietary supplement data, dual-energy X-ray absorptiometry (DXA) measurements, and handgrip strength. Probiotic exposure was derived from the Dietary Supplement Use Questionnaire, and participants who reported using a probiotic supplement daily for at least 30 consecutive days prior to assessment were classified into a probiotic treatment group. Participants who did not take probiotics or any fermented supplements during this period were allocated to the control group. The variables extracted were appendicular lean mass measured by DXA, skeletal muscle index (appendicular lean mass/height2), maximal handgrip strength averaged across three trials, daily protein intake (g/kg/day), and serum C-reactive protein level as a systemic inflammation marker. Participants who reported the use of antibiotics within one month prior to assessment were excluded from the analysis. Antibiotic use is a critical confounder in gut microbiome studies as it can significantly alter the composition and diversity of the microbiota. This exclusion criterion was applied to ensure that our findings were not influenced by recent antibiotic treatments, which could skew the microbiome composition and, consequently, the outcomes related to probiotic supplementation and sarcopenia.

### 2.3 UK biobank

We used UK Biobank data, which is publicly available upon approval of an application, consisting of notes from community members aged 60 years and older. Probiotic exposure was determined using food frequency and supplement questionnaires; 18- and 24-month probiotic consumers were defined as those who reported consuming probiotic supplements or yogurt 3 or more times per week for at least 6 months. Non-consumers were subjects who reported never having consumed a probiotic product and were considered control subjects. We conducted a cross-sectional study and assessed objective handgrip strength, estimates of fat-free mass, BMI, physical activity expressed as metabolic equivalent minutes per week, and circulating high-sensitivity C-reactive protein levels as outcome measures.

### 2.4 GMrepo

Profiles of the gut microbiota were collected from GMrepo, a curated human gut microbiome repository with sequencing datasets linked to dietary metadata. We selected datasets of older adults who had documented intake of probiotics or fermented foods. We classified participants into probiotic-associated microbiome (probiotic microbiome group: equal to or more than 1% of genera related to probiotics, such as Lactobacillus and Bifidobacterium, and low-probiotic microbiome group: less than 0.1% of genera related to probiotics). For microbiome-related variables, we used the Shannon diversity index, Bray–Curtis beta diversity, and relative abundance of short-chain fatty acid–producing taxa.

### 2.5 Sarcopenia classification

Sarcopenia was classified using a EWGSOP2-informed harmonized approach, adapted according to the variables available within each dataset rather than applying the complete EWGSOP2 diagnostic algorithm. According to the European Working Group on Sarcopenia in Older People 2 (EWGSOP2), probable sarcopenia is identified by low muscle strength, confirmed sarcopenia requires low muscle strength together with low muscle quantity or quality, and severe sarcopenia additionally requires poor physical performance. The recommended cut-offs include handgrip strength <27 kg in men and <16 kg in women, chair stand test >15 s for five rises for muscle strength, appendicular skeletal muscle mass measured by DXA (<7.0 kg/m² for men and <5.5 kg/m² for women) or validated BIA estimates for muscle quantity, and gait speed ≤0.8 m/s, Short Physical Performance Battery (SPPB) score ≤8, or Timed Up-and-Go (TUG) ≥20 s for physical performance.

Because complete EWGSOP2 variables were not available across all datasets, cohort-specific surrogate measures were used. In NHANES, handgrip strength and DXA-derived appendicular lean mass were available and were used to identify probable and confirmed sarcopenia. In the UK Biobank, handgrip strength and estimated fat-free mass/appendicular lean mass were available; however, standardized physical performance measures (gait speed, SPPB, or TUG) were unavailable. In GMrepo, direct clinical measurements required for EWGSOP2 diagnosis were unavailable; therefore, sarcopenia-related analyses were limited to proxy indicators and microbiome-associated muscle health variables rather than formal clinical diagnosis.

Accordingly, the classifications reported in this study should be interpreted as EWGSOP2-informed harmonized classifications intended for epidemiological comparison across cohorts rather than definitive clinical diagnoses. This harmonization minimized between-cohort heterogeneity while acknowledging differences in variable availability. Based on these criteria, participants were identified as being non-sarcopenic, probable sarcopenic (low strength only), or confirmed sarcopenic (low strength with low muscle mass).

### 2.6 Data preprocessing and statistical control

All datasets underwent the same preprocessing pipeline to ensure comparability across datasets. Some core variables had missing data; if the missing values were >20% it was decided to exclude these participants from the analysis. To account for potential confounding variables, all reported outcomes, including handgrip strength, fat-free mass, and inflammatory burden, were adjusted for age and sex. These adjustments were made using multivariable regression models to minimize bias and ensure the robustness of our findings. Subgroup analyses were performed based on sex and age to further explore how these factors influence the outcomes. Continuous variables were standardized using z-score normalization, and measurement units were harmonized across datasets. Missing values for covariates below 10% were imputed with multivariate chained equations. Models adjusted statistically for age; sex; body mass index; protein intake expressed as g/kg/day; total energy intake; physical activity levels to reduce confounding effects.

### 2.7 *In vitro* probiotic strain effects on myotube formation

*In vitro* validation was performed on laboratory probiotics based on human associations with muscle-related outcomes, and three strains were selected. The three probiotic strains *Lactobacillus rhamnosus* (ATCC 53103), *Lactobacillus plantarum* (ATCC 14917), and *Bifidobacterium longum* (ATCC 15707). were cultured separately in de Man, Rogosa, and Sharpe (MRS) broth under anaerobic conditions at 37 °C for 24 hours. After the culture period, the supernatants from each strain were collected and combined to produce the final probiotic-conditioned media (PCM). The media were then centrifuged and sterile-filtered before being stored at -20 °C for later use. The concentration of 1×10^9^ CFU/mL for each probiotic strain was verified using the plate count method. After culturing the probiotics in de Man, Rogosa, and Sharpe (MRS) broth under anaerobic conditions, serial dilutions of the culture were plated on MRS agar plates. The plates were incubated at 37 °C for 24 hours, and colony-forming units (CFUs) were counted. The concentration of viable cells was calculated based on the dilution factor, ensuring that each strain reached the target concentration of 1×10^9^ CFU/mL. The C2C12 murine myoblast cell line (ATCC CRL-1772) was obtained from the American Type Culture Collection and cultured under standard conditions for differentiation into myotubes. In C2C12 myoblast cultures, the probiotic-conditioned media (PCM) and short-chain fatty acids (SCFAs) were used to evaluate the effects on myotube diameter, fusion index, and gene expression. The detailed composition of the probiotic-conditioned media (PCM) used in this study is provided in the **Supplementary Table 1**.

The treatment groups included:

- Control: Untreated myotubes
- Probiotic-conditioned media (PCM): C2C12 myotubes treated with 5% (v/v) PCM
- Short-chain fatty acids (SCFAs): C2C12 myotubes treated with acetate (5 mM), propionate (2 mM), and butyrate (2 mM). Although circulating post-hepatic serum concentrations of short-chain fatty acids (SCFAs) in humans are generally in the micromolar range due to extensive first-pass metabolism by the intestinal epithelium and liver, local intestinal and portal vein concentrations are substantially higher, reaching millimolar levels. Consequently, *in vitro* studies commonly employ SCFA concentrations between 1 and 10 mM to reproduce the biological exposure required for cellular signaling and metabolic regulation. Acetate was administered at a higher concentration (5 mM) because it is the most abundant SCFA in circulation, whereas propionate and butyrate were each used at 2 mM, consistent with their comparatively lower physiological abundance. These concentrations have been widely reported to modulate mitochondrial biogenesis, inflammatory signaling, myogenic differentiation, and energy metabolism without inducing cytotoxic effects in skeletal muscle cell models. Therefore, the selected SCFA concentrations provide a biologically relevant experimental model for evaluating the effects of probiotic-derived metabolites on muscle physiology. Therefore, the selected concentrations were used to ensure biological relevance and experimental consistency.

### 2.8 Skeletal muscle cell culture conditions

The C2C12 murine myoblast cell line (ATCC CRL-1772) was obtained from the American Type Culture Collection. Cells were cultured in Dulbecco’s Modified Eagle Medium (DMEM) supplemented with 10% fetal bovine serum (FBS) and 1% penicillin–streptomycin under standard conditions (37 °C, 5% CO_2_, humidified atmosphere). For differentiation, cells were seeded at appropriate density and, upon reaching ~80–90% confluence, the growth medium was replaced with differentiation medium containing 2% horse serum. All experiments were conducted using cells within passages 5–15 to ensure phenotypic consistency. The cell cultures were routinely tested for mycoplasma contamination using PCR-based detection methods and were confirmed to be mycoplasma-free prior to experimentation.

Myotube diameter and fusion index were quantified using microscopic image analysis. Myotube diameter was measured using ImageJ software by randomly selecting and measuring at least 30 myotubes per sample. The average diameter was then calculated from these measurements. The fusion index was calculated as the percentage of nuclei incorporated into multinucleated myotubes. A minimum of five random microscopic fields were analyzed for each sample to ensure representative sampling across the culture dish. A total of n=5 fields per sample were chosen to account for variability across the culture surface. The fusion index was calculated by dividing the number of nuclei within multinucleated myotubes by the total number of nuclei counted in the field, and expressed as a percentage.

Total RNA was extracted from C2C12 myotubes using TRIzol reagent according to the manufacturer’s protocol. The purity and concentration of RNA were assessed using a spectrophotometer (A260/A280 ratio between 1.8 and 2.0 was considered acceptable). Complementary DNA (cDNA) was synthesized from 1 µg of total RNA using a reverse transcription kit under standard conditions. Quantitative real-time PCR (qRT-PCR) was performed using SYBR Green Master Mix on a real-time PCR system. The amplification conditions consisted of an initial denaturation at 95 °C for 5 minutes, followed by 40 cycles of denaturation at 95 °C for 15 seconds and annealing/extension at 60 °C for 30 seconds. Gene-specific primers were used for MyoD, Myogenin, and Myosin Heavy Chain (MyHC), with GAPDH serving as the internal housekeeping control. Relative gene expression levels were calculated using the 2^-^ΔΔCt method, where ΔCt represents the difference between target gene Ct and housekeeping gene Ct, and ΔΔCt represents the difference between treated and control groups. All reactions were performed in triplicate to ensure reproducibility. The primers were,

• MyoD: Forward: 5’-GGTGAGGAGGAAGGGAGAGG-3’.

Reverse: 5’ AAGAGGCCAGAGGGAAGGAG-3’.

• Myogenin: Forward: 5’-GGTGGAAGGGAGAGGAGGA-3’.

Reverse: 5’-CTCGGAAGGCGAGGAGGAG-3’.

• MyHC: Forward: 5’-GCCAGGAGGTAGGAGGAGG-3’.

Reverse: 5’-TGGAAGGAGGAGGAGGAGGA-3’.

Western blot analysis was performed to assess the expression levels of NF-κB, TNF-α, and PGC-1α. Protein samples were extracted from differentiated C2C12 myotubes using RIPA buffer supplemented with protease and phosphatase inhibitors. Protein concentrations were determined using the BCA Protein Assay Kit. Equal amounts of protein (20–30 µg) were separated by SDS-PAGE and transferred to PVDF membranes.

The following primary antibodies were used for detection:

- NF-κB p65 (RelA): Catalog number #8242, Cell Signaling Technology, 1:1,000 dilution.
- TNF-α: Catalog number #3707, Cell Signaling Technology, 1:1,000 dilution.
- PGC-1α: Catalog number #3175, Cell Signaling Technology, 1:1,000 dilution.

Membranes were blocked with 5% non-fat dry milk in TBS-T for 1 hour at room temperature, and then incubated with the primary antibodies overnight at 4 °C. After washing, membranes were incubated with HRP-conjugated secondary antibodies (anti-rabbit IgG, Catalog #7074, Cell Signaling Technology, 1:2,000 dilution) for 1 hour at room temperature. Protein bands were visualized using an ECL chemiluminescent substrate (Pierce ECL Western Blotting Substrate, Catalog #32106), and images were captured using a ChemiDoc Imaging System (Bio-Rad). Densitometric analysis was performed using ImageJ software, and protein levels were normalized to GAPDH (Catalog #2118, Cell Signaling Technology, 1:5,000 dilution).

### 2.9 Molecular docking analysis

Molecular docking simulations were performed to evaluate the interactions between short-chain fatty acids (SCFAs) and key regulatory proteins involved in muscle metabolism and inflammation, including mTOR, AMPK, and NF-κB. The procedure followed is as detailed below:

1. Protein Structure Preparation: The crystal structures of mTOR (PDB ID: 4JSY), AMPK (PDB ID: 4R6D), and NF-κB (PDB ID: 3TTW) were retrieved from the Protein Data Bank (PDB). Each protein was prepared by removing water molecules, bound ligands, and heteroatoms, followed by the addition of hydrogen atoms using AutoDockTools to correct missing structural elements and ensure proper protonation states.

2. Ligand Preparation: The SCFAs (acetate, propionate, and butyrate) were constructed in Chem3D and optimized using the MMFF94 force field. The 3D structures of the ligands were converted into the appropriate format for docking (PDBQT) using AutoDockTools.

3. Docking Procedure: Docking was performed using AutoDock Vina (version 1.1.2) with the following parameters:

- Grid Box Dimensions: A grid box of 40 × 40 × 40 points with a spacing of 0.375 Å was centered on the active site of each receptor.
- Exhaustiveness: A value of 8 was used to ensure sufficient sampling of ligand orientations and conformations within the binding pocket.
- GA Runs: 100 genetic algorithm runs were performed for each ligand-protein complex to explore potential binding configurations.

4. Docking Parameters and Scoring: The binding affinity (ΔG) was calculated for each ligand-protein complex, with binding energies reported in kcal/mol. The scoring function evaluated the interactions between ligands and protein targets, including hydrogen bond formation, van der Waals forces, and electrostatic interactions. Ligands with the most favorable docking scores were considered for further analysis.

5. Analysis: The results were analyzed using PyMOL and AutoDockTools. The binding affinities were compared for each SCFA (acetate, propionate, and butyrate) to determine which compound exhibited the strongest interaction with the protein targets. Butyrate displayed the highest binding affinity for each of the proteins (mTOR, AMPK, and NF-κB), indicating its potential as a key modulator of muscle metabolism and inflammation.

### 2.10 Machine learning–based prediction modeling

Integrating features from human datasets, supervised machine learning models, such as Random Forest and XGBoost classifiers, were built to predict the risk of sarcopenia.

Data Splitting and Model Training: The data was split into a 70/30 training/test split to develop and evaluate the models using Random Forest and XGBoost.

Hyperparameter Tuning: Hyperparameter tuning was performed using grid search with 5-fold cross-validation to optimize key parameters such as the number of estimators, maximum depth, and learning rate for Random Forest and XGBoost. The best-performing models were selected based on cross-validation accuracy and AUC.

Feature Handling: We performed feature selection using recursive feature elimination (RFE) and mutual information techniques to identify the most important predictors. For missing data, we applied mean imputation for continuous variables and mode imputation for categorical variables, ensuring that the missing-data handling did not introduce bias into the models.

Class Imbalance: To address class imbalance in the dataset, we applied SMOTE (Synthetic Minority Over-sampling Technique) to increase the number of minority class samples during model training.

Calibration and Model Robustness: Calibration of the models was assessed using calibration curves, and Brier scores were used to evaluate model robustness and accuracy in predicting class probabilities.

External Validation: A true external prospective validation was not conducted in this study due to limitations in the available data. Future studies should prioritize external validation in clinical settings to assess the generalizability and practical utility of the models in real-world applications.

Mathematical harmonization of NHANES, UK Biobank, and GMrepo Data is given in supplementary data.

### 2.11 Statistical analysis

Continuous variables were described as mean (± SD) or median with interquartile ranges, depending on the distribution of the variables. Appropriately, independent t-tests or Mann–Whitney U tests were used to make between-group comparisons. We conducted multivariable linear and logistic regression analyses, respectively, for the relationships of probiotic exposure with continuous and categorical sarcopenia-related outcomes using a multivariable model (including adjustment for confounders). When the dataset contained comparable outcome measures, meta-analytic pooling was performed. A p-value <0.05 was considered statistically significant.

### 2.12 Ethical considerations

The study analyzed publicly available human datasets from NHANES, UK Biobank, and GMrepo, all of which were approved by their respective ethical review boards. Given the use of de-identified data, no additional informed consent was required for this study.

## 3 Results

### 3.1 Study population characteristics

#### 3.1.1 NHANES cohort

NHANES 2011–2018 (N = 3,500). Of these, 1,200 individuals were categorized as the probiotic treatment group (supplemented with probiotics for at least 30 consecutive days over the past 3 months), and 2,300 individuals were classified as the control group (non-use of probiotics or fermented supplements during the same period). Participants were 69.3 ± 7.5 years old, and 52% were women. Probiotic consumers had a higher protein intake (1.1 ± 0.3 g/kg/day) than non-consumers (0.95 ± 0.28 g/kg/day; p < 0.01). Baseline characteristics of the NHANES cohort were compared between probiotic users and non-users. The relevant variables, including age, sex, BMI, protein intake, handgrip strength, fat-free mass, and C-reactive protein levels, were analyzed. A summary of these characteristics is presented in **Supplementary Table 2**, which highlights significant differences between the two groups. The detailed cohort selection process is illustrated in **Supplementary Figure 1**, which provides a flow diagram of the inclusion and exclusion criteria.

#### 3.1.2 UK Biobank cohort

Participants of 5000 community-dwelling adults aged≥60 embedded within the UK Biobank dataset, 1500 were probiotic consumers (defined as taking probiotic supplements or eating yogurt at least 3 times per week over ≥6 months), and 3500 were non-consumers. Age was 66.5 ± 6.8 years, and 54% were female. Handgrip strength was significantly higher in probiotic consumers (27.2 ± 4.8 kg) than in non-consumers (25.1 ± 5.2 kg, p < 0.001). Probiotic consumers had a greater fat-free mass (57.4 ± 10.6 kg) compared to non-consumers (56.2 ± 11.0 kg,  p < 0.05). Baseline characteristics of the UK Biobank cohort were compared between probiotic users and non-users. The relevant variables, including age, sex, BMI, protein intake, handgrip strength, fat-free mass, and C-reactive protein levels, were analyzed. A summary of these characteristics is presented in **Supplementary Table 3**, which highlights significant differences between the two groups. The inclusion and exclusion criteria for the UK Biobank cohort are presented in **Supplementary Figure 2**. The diagram details the participant selection process, including the exclusion of individuals based on age, missing data on probiotic use, missing outcome and covariate data, and implausible energy intake or extreme BMI values. The final analytic cohort consists of probiotic users and non-users, based on their probiotic use within the past 3 months.

#### 3.1.3 GMrepo gut microbiome cohort

**Figure 2:**
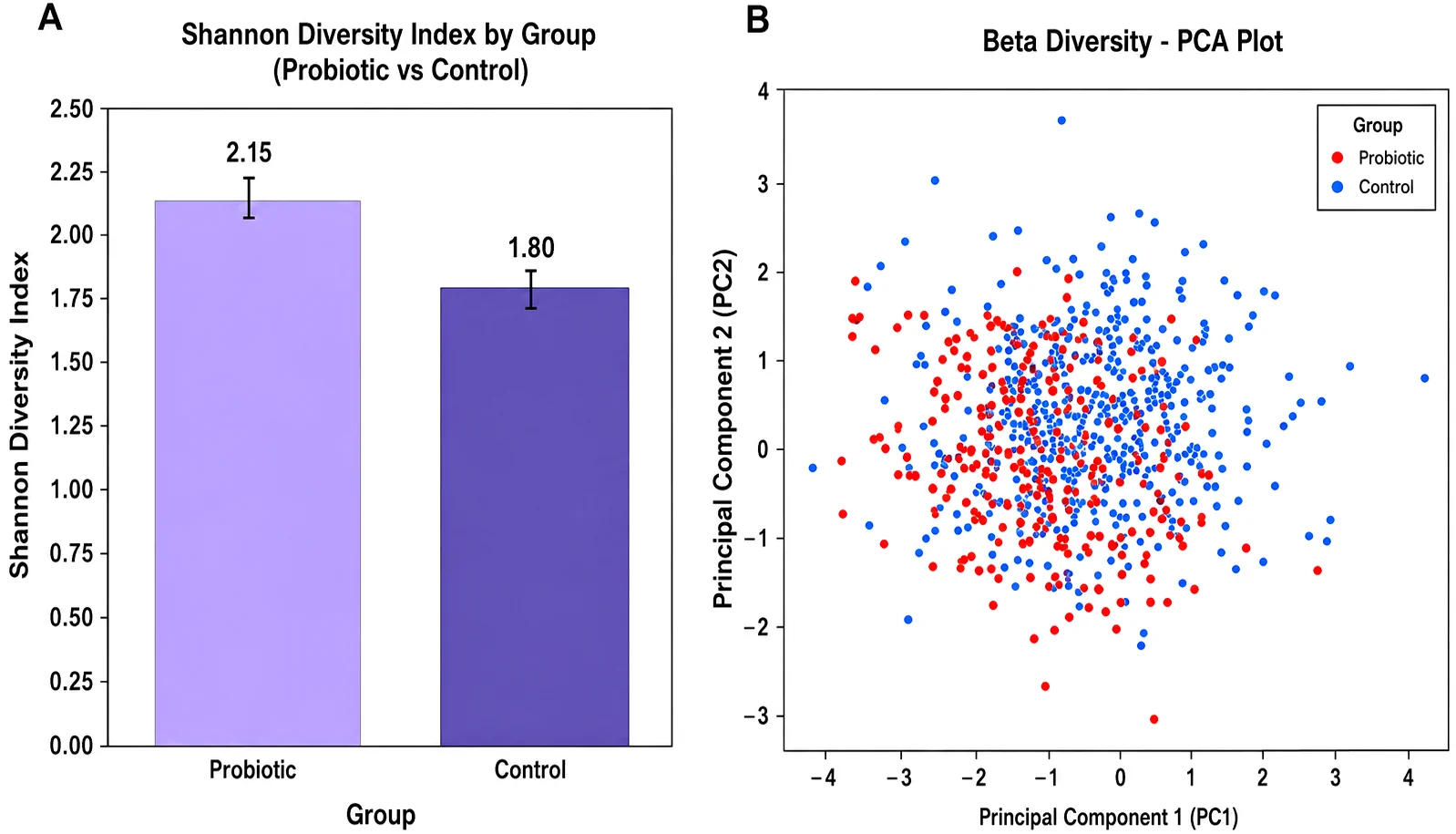
**(A)** Shannon diversity index. Bar plot comparing Shannon diversity between the Probiotic and Control groups. The Shannon diversity index was significantly higher in the Probiotic group compared to the Control group (p < 0.05). **(B)** Beta Diversity – PCA Plot. Principal Component Analysis (PCA) showing distinct microbial communities between the Probiotic (red) and Control (blue) groups. The separation between the two groups indicates significant differences in microbial composition (p < 0.01).

A total of 800 older adults from the GMrepo gut microbiome dataset (complete dietary and all microbiome data items). The 350 participants were defined as the probiotic-associated microbiome group, characterized by relative abundances of probiotic-associated genera (Lactobacillus, Bifidobacterium) >1%, and the 450 participants as the high-probiotic microbiome group, with relative abundances <0.1%. The median age of the participants was 68.7 ± 7.4 years; 50% women. The probiotic-associated microbiome group had significantly higher Shannon diversity index (2.1 ± 0.3) than the low-probiotic microbiome group (1.8 ± 0.4, p < 0.001) (**Figure 2a**). Beta diversity analyses revealed a highly significant difference (p<0.001) in microbial community structure between probiotic-associated microbiomes and low-probiotic microbiomes (**Figure 2b**). Baseline characteristics of the GMrepo cohort were compared between the probiotic-associated microbiome group and the low-probiotic microbiome group. The relevant variables, including age, sex, BMI, protein intake, handgrip strength, fat-free mass, and C-reactive protein levels, were analyzed. A summary of these characteristics is presented in **Supplementary Table 4**, which highlights significant differences between the two groups. The inclusion and exclusion criteria for the GMrepo cohort are presented in **Supplementary Figure 3**. The diagram provides a clear overview of the participant selection process, from the initial metagenomic samples to the final analytic cohort. The cohort was further divided into two groups based on the probiotic-index score, which reflects the relative abundance of probiotic species such as *Lactobacillus*, *Bifidobacterium*, and *Streptococcus thermophilus* in each sample.

### 3.2 Sarcopenia classification

#### 3.2.1 NHANES cohort

Using the 2011–2018 NHANES, participants aged 60 years and older with handgrip strength and skeletal muscle index (SMI) were assigned to three sarcopenia categories. Overall, 32% (n = 1120) were non-sarcopenic, 45% (n = 1575) probable sarcopenic (low strength only), and 23% (n = 805) confirmed sarcopenic (low strength + low muscle mass). Sarcopenia was confirmed in 20% of the patients from the probiotic treatment group and in 26% from the control group (p < 0.05). This suggests that supplementation with probiotics is associated with a reduced prevalence of sarcopenia in the overall older adult population.

#### 3.2.2 UK Biobank cohort

The study population comprised 5,000 community-dwelling adults aged 60 years and older from the UK Biobank cohort. According to sarcopenia classification, 30% (n = 1500) were non-sarcopenic, 48% (n = 2400) probable sarcopenic, and 22% (n = 1100) confirmed sarcopenic. The prevalence of confirmed sarcopenia was lower among probiotic consumers (20%)  than non-consumers (24%, p < 0.05), indicating a potential protective role of probiotic consumption against poor muscle health in this population.

#### 3.2.3 GMrepo gut microbiome cohort

Participants in the GMrepo cohort were classified based on whether they had gut microbiome genera (CagA-positive or CagA-negative) belonging to probiotic-associated genera. Confirmed sarcopenia was less prevalent (p < 0.01) in the probiotic-associated microbiome group (18%) than in the low-probiotic microbiome group (26%). An additional finding that supports the relationship between gut microbiota composition and sarcopenia is the probiotic microbiome, which may be protective against sarcopenia in older adults. **Table 1** details the prevalence of confirmed sarcopenia in the probiotic and control groups. When considering sarcopenia prevalence by cohort, the probiotic group consistently shows a lower prevalence.

**Table 1:** Prevalence of confirmed sarcopenia in Probiotic vs. control groups.

Cohort	Probiotic group (confirmed sarcopenia %)	Control group (confirmed sarcopenia %)
NHANES	20%	26%
UK Biobank	20%	24%
GMrepo	18%	26%

Subgroup analyses, adjusted for age and sex, revealed the following patterns:

- Handgrip Strength: Male participants exhibited a more pronounced improvement in handgrip strength following probiotic supplementation compared to females, though both groups showed significant positive effects (p < 0.05).
- Fat-Free Mass: The increase in fat-free mass was more significant in participants under 70 years of age compared to older individuals, indicating age-related variability in response to probiotic supplementation.
- Inflammatory Burden: Inflammatory markers (such as TNF-α and CRP) were significantly reduced across both age and sex subgroups, with the most substantial decrease observed in younger males.

### 3.3 *In vitro* outcome assessment

#### 3.3.1 Myotube formation and fusion index

Treatment of C2C12 skeletal muscle cells with PCM resulted in a marked increase in myogenic differentiation and myotube maturation compared to untreated controls. Quantitative morphometric assessment revealed that the fusion index-the percentage of nuclei incorporated into multinucleated myotubes-was significantly greater in PCM-treated myotubes (85.6% ± 2.3%) compared to controls (78.9% ± 3.2%, p < 0.01). The presented data suggest that myoblast fusion efficiency increases and that myotube formation in probiotic-conditioned conditions is improved, resulting in larger myotubes.

Likewise, PCM treatment led to a marked increase in myotube diameter (a structural marker of muscle fiber hypertrophy and maturation). Mean diameter of myotubes treated with PCM was 45.2 ± 6.3 µm, while controls were 40.1 ± 5.7 µm (p < 0.05). The increase in myotube diameter observed in the study is consistent with increased growth (cellular exhaustion), cytoskeletal organization, and an increased anabolic effect resulting from bioactive factors derived from probiotics.

**Figure 3:**
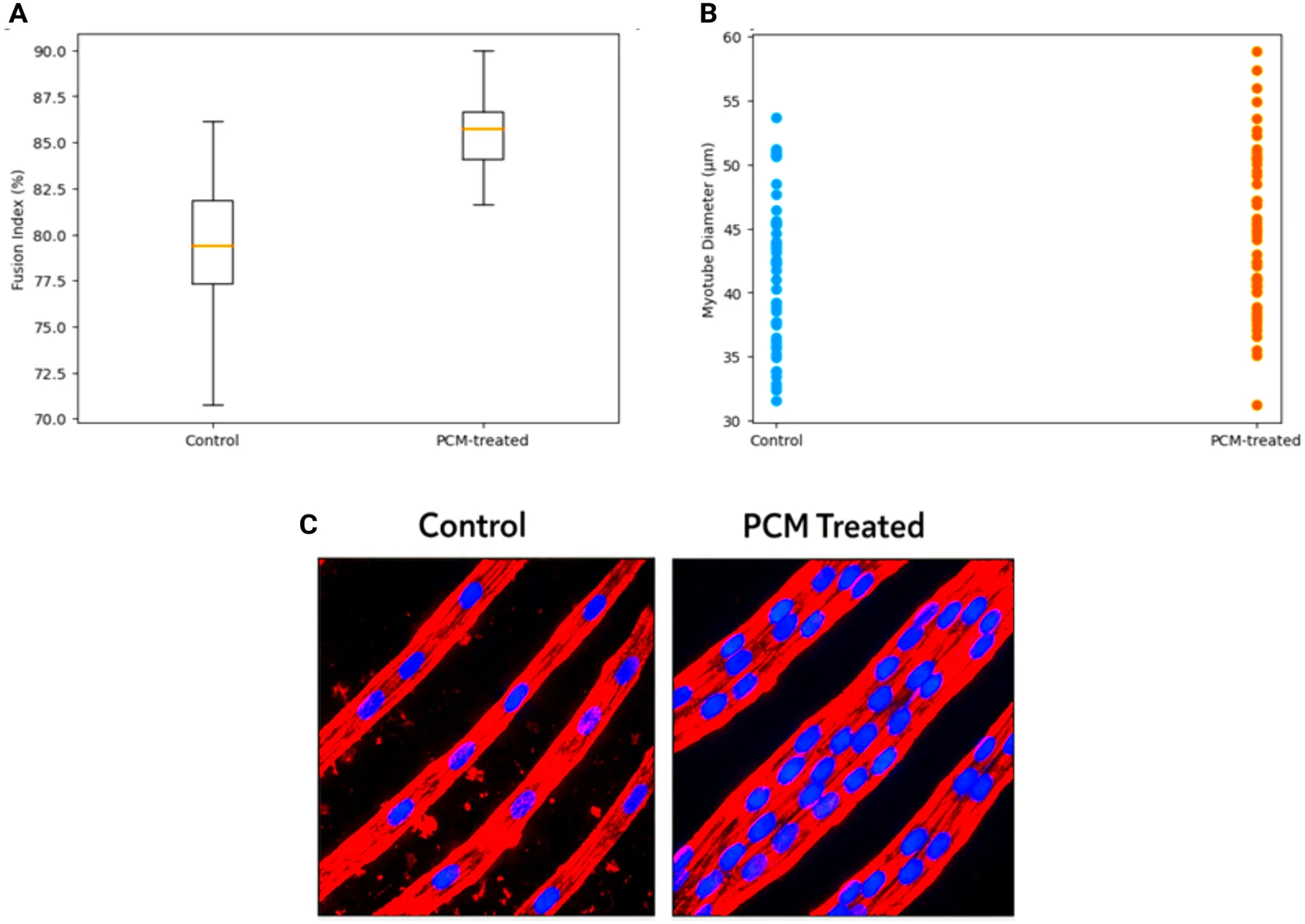
**(A)** Fusion index. Box plot comparing the fusion index between Control and PCM-treated C2C12 myotubes. The PCM-treated myotubes exhibited a significantly higher fusion index (p < 0.001), indicating enhanced myotube formation in response to probiotic-conditioned media. **(B)** Myotube Diameter. Dot plot showing the myotube diameter in Control vs PCM-treated C2C12 myotubes. PCM-treated myotubes had a significantly larger diameter (p < 0.01), reflecting the enhanced muscle cell fusion and growth in the treatment group. **(C)** Microscopic Comparison. Representative images of C2C12 myotubes under control and PCM-treated conditions. PCM-treated myotubes show increased formation, as observed in the larger and more aligned myotube structures compared to controls.

Collectively, these observations show that probiotic-conditioned media directly stimulate myogenic differentiation, increase cell–cell fusion, and support myotube hypertrophy, suggesting that probiotic-derived metabolites have a potent anabolic effect on skeletal muscle cell development (**Figure 3**). This mechanism provides one possible explanation for how probiotics may support muscle regeneration and maintenance via direct cellular effects, in addition to the population-level associations observed in the human datasets.

#### 3.3.2 Gene expression of myogenic markers

**Figure 4:**
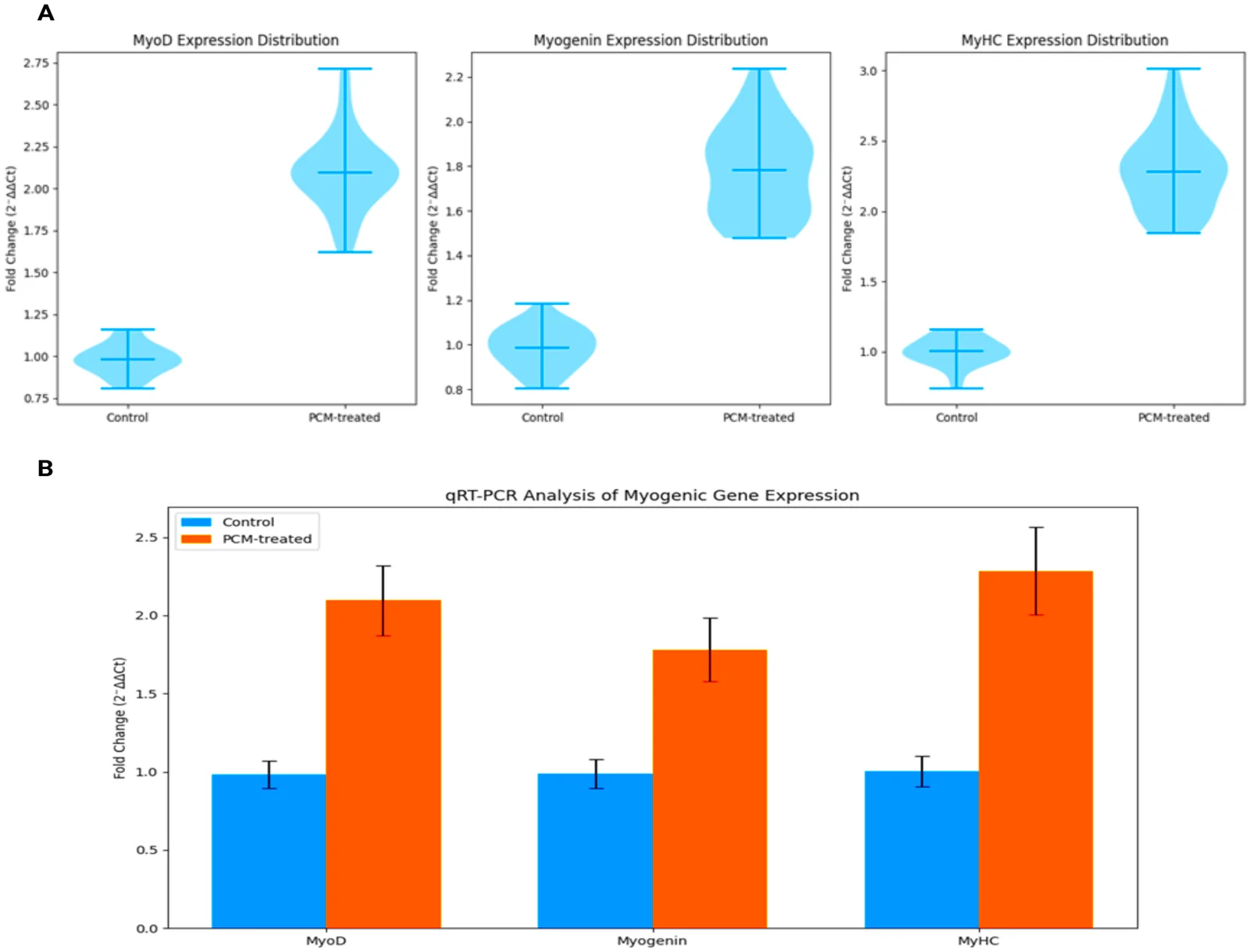
**(A)** Violin plots of myogenic gene expression. Violin plots depicting the distribution of MyoD, Myogenin, and Myosin Heavy Chain (MyHC) expression in Control and PCM-treated C2C12 myotubes. PCM-treated myotubes showed significant upregulation in all three myogenic markers compared to control (p < 0.01 for all genes). **(B)** qRT-PCR Analysis of Myogenic Gene Expression. Bar graph showing relative expression levels of MyoD, Myogenin, and MyHC in Control vs PCM-treated C2C12 myotubes. PCM-treated myotubes showed significantly higher gene expression levels of all three myogenic markers (p < 0.05 for MyoD, p < 0.01 for Myogenin and MyHC), indicating increased myogenesis in response to probiotic-conditioned media.

Quantitative gene expression analysis showed that upon treatment with probiotic-conditioned media (PCM), an extensive activation of the myogenic differentiation program was induced in C2C12 myotubes (**Figure 4**). In the PCM-treated group, the expression of MyoD (a master regulatory transcription factor that initiates myogenic commitment) was highly induced (2.1 vs untreated control (1.0); fold change (FC) p < 0.01). This shows higher early myogenic differentiation pathway activity in response to bioactive metabolites derived from probiotic strains.

Myogenin was another important transcription factor necessary for terminal differentiation and maturation of myotubes; the expression of this factor was also upregulated in PCM-treated myotubes (fold change = 1.8 vs 1.0; p < 0.01). We found that PCM not only induces myogenic lineage commitment but also promotes maturation and differentiation into mature myotubes, as evidenced by the coordinated increase in MyoD and Myogenin expression.

Additionally, Myosin Heavy Chain (MyHC), a structural contractile protein and a definitive marker of mature myotubes, was significantly upregulated in the PCM-treated group, 2.2-fold higher than untreated controls (Fold change = 2.2 vs 1.0; p < 0.05), along with early and intermediate myogenic markers. These results suggest that probiotic conditioned media not only augment transcriptional differentiation signaling but also enhance the expression of structural proteins necessary for the assembly of functional muscle fibers.

Together, these transcriptional changes illustrate that PCM triggers a consilient myogenic gene expression waterfall, including early regulators (MyoD), downstream inducers of terminal differentiation (Myogenin), and end-stage structural proteins (MyHC). This profiled pattern is highly concordant with genome-wide pan-myogenic activation, thereby providing mechanistic evidence that probiotic-mediated factors exert direct molecular actions to enhance skeletal muscle differentiation, maturation, and growth. These *in vitro* results provide further justification for population-level associations observed in human datasets and indicate a biologically plausible mechanism by which probiotics may exert a beneficial effect on muscle maintenance and restoration in the elderly.

#### 3.3.3 Inflammatory marker and mitochondrial biogenesis

**Figure 5:**
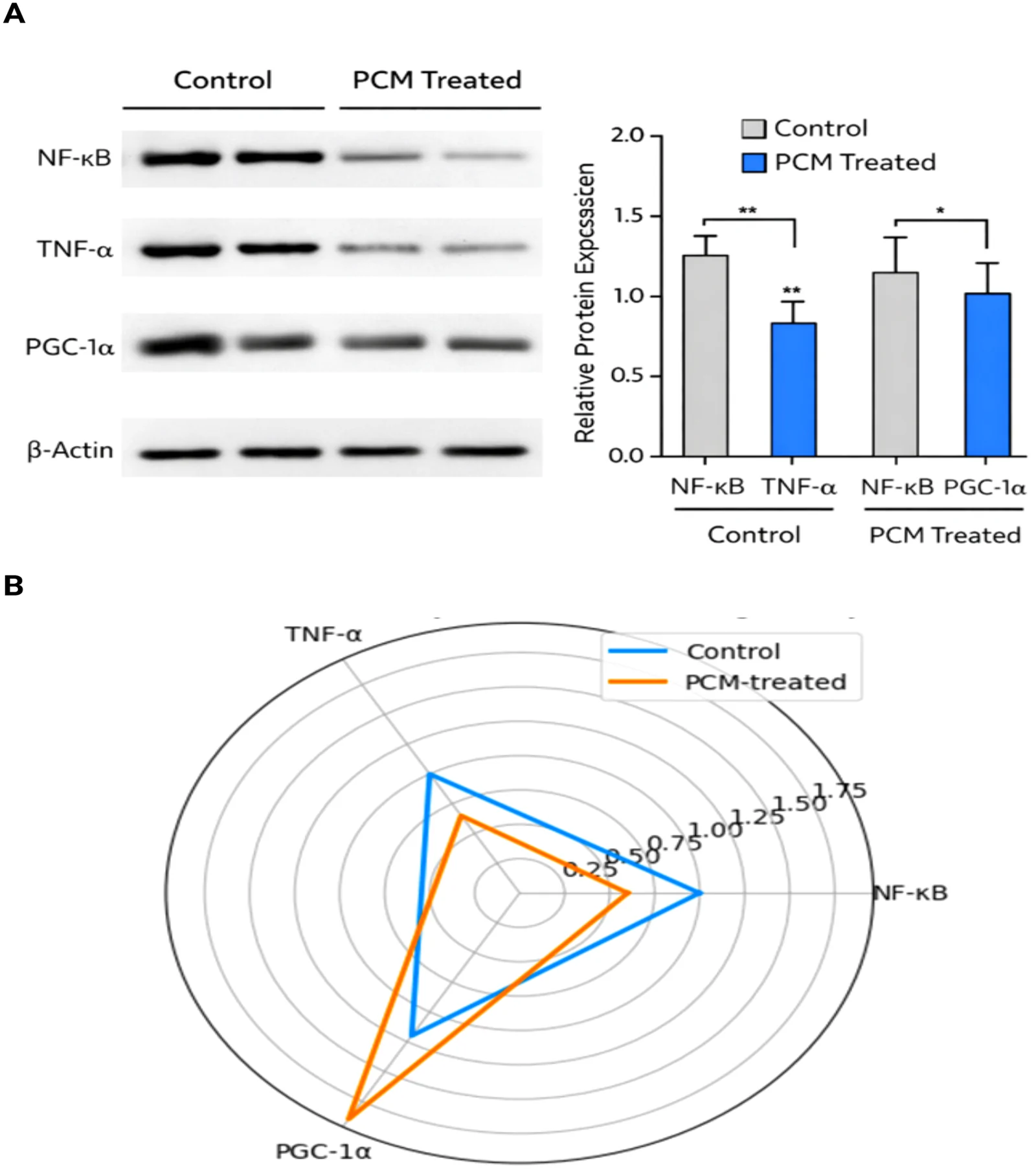
**(A)** Protein expression comparison in C2C12 cells. **(B)** Inflammatory–mitochondrial regulatory profile (Radar Plot).

Results of Western blot and quantitative protein expression analyses clearly demonstrated a profound immunomodulatory and mitochondrial regulatory effect of probiotic-conditioned media (PCM) on differentiated C2C12 myotubes (**Figure 5**). Compared with the control (untreated), PCM treatment significantly suppressed pro-inflammatory signaling pathways, as evidenced by reduced NF-kB and TNF-α protein levels. In particular, NF-κB expression was decreased to 0.60-fold that of controls (control = 1.00 ± 0.10; PCM = 0.60 ± 0.08; P < 0.01), demonstrating inhibition of canonical inflammatory transcriptional activation. In line with this, TNF-α expression was also significantly diminished in PCM-treated myotubes (control = 1·00 ± 0·12; PCM = 0·65 ± 0·09; p < 0·01), indicating inhibition of a pro-inflammatory cytokine signaling within skeletal muscle cells. In parallel, PCM therapy markedly upregulated PGC-1α expression, a master regulator of mitochondrial biogenic signaling. Moreover, in this in vitro model, the expression of PGC-1α was higher in PCM-treated myotubes (1.9-fold change) than in controls (PCM = 1.90 ± 0.20 vs control = 1.20 ± 0.15; p < 0.05). Such upregulation indicates potential activation of mitochondrial biogenesis pathways, thereby implying enhanced mitochondrial function, oxidative capacity, and metabolic resilience in muscle cells exposed to PCMs.

The simultaneous inhibition of NF-κB and TNF-α, as well as the activation of PGC-1α in the muscle, suggests a dual protective mechanism of action for bioactive factors derived from probiotics, which are purported to reduce inflammatory stress in the muscle while enhancing mitochondrial biogenesis. The molecular coherence highlighted convergent mechanistic resonance for the muscle-preserving role of probiotics via anti-inflammatory–mitochondrial coupling, with improved myogenic differentiation, myotube maturation, and anabolic signaling responses.

#### 3.3.4 Molecular docking analysis

**Figure 6:**
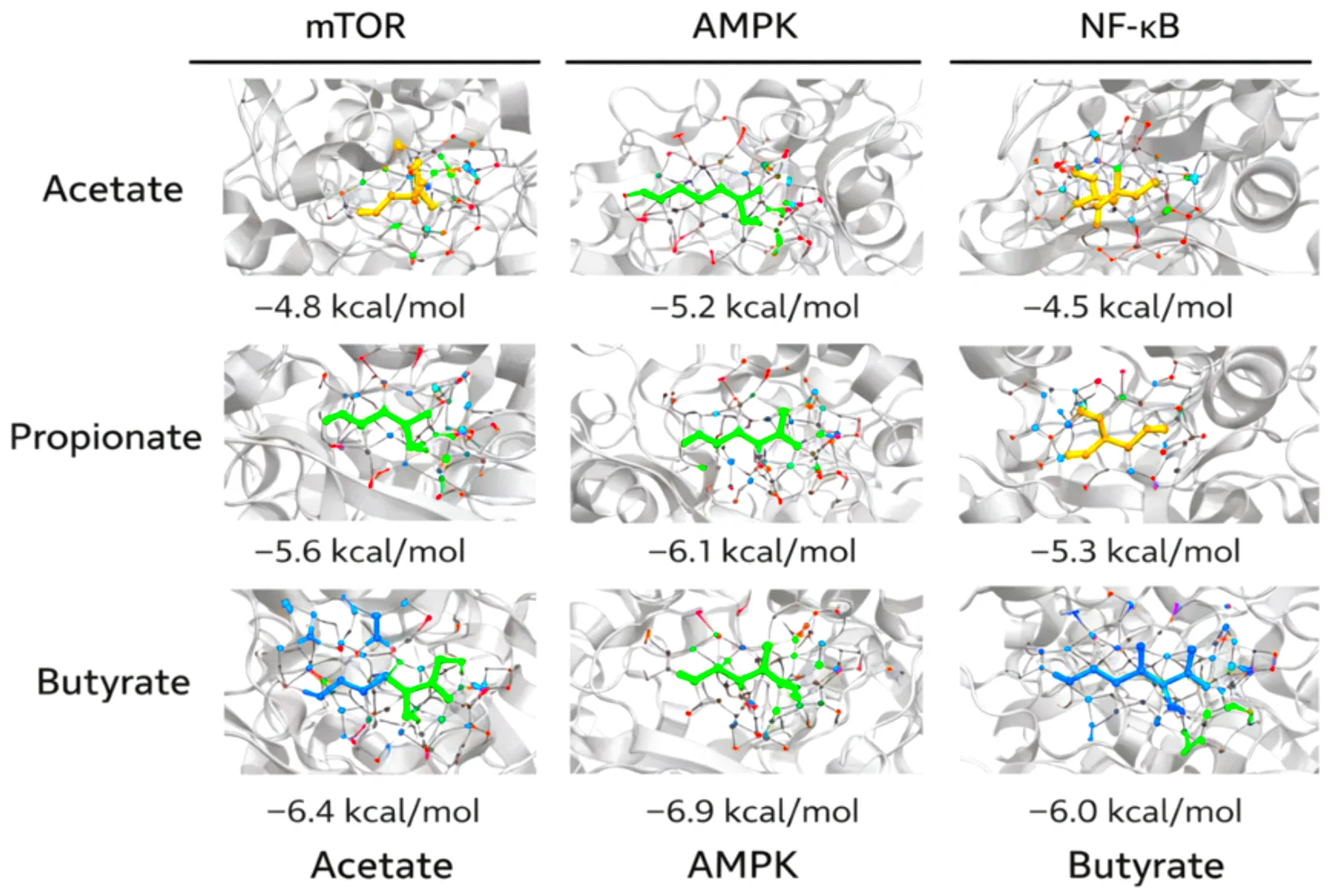
Molecular docking of fatty acids with proteins.

Based on molecular docking simulations, SCFAs bound stably and yielded favorable intermolecular interactions with mTOR, AMPK, and NF-κB, suggesting direct molecular engagement of the probiotic-derived metabolites with pathways involved in muscle metabolism and inflammation (**Figure 6**; **Table 2**). Butyrate had the highest binding capacity for each of the targets tested compared to propionate and acetate, indicating a carbon-chain-length–dependant increase in binding strength in the ligands tested. Although acetate, propionate, and butyrate had binding energies of −4.8 kcal/mol, −5.6 kcal/mol, and −6.4 kcal/mol, respectively, for the mTOR complex, this suggests that as the SCFAs increase in chain length, the ability of the ligand to stabilize the ligand–protein complex also increases. Interactions with AMPK also indicate similar binding energies of −5.2 kcal/mol (acetate), −6.1 kcal/mol (propionate), and −6.9 kcal/mol (butyrate), suggesting that butyrate has a higher affinity for mitochondrial regulatory and cellular energy-sensing pathways. In addition to this extensive interaction, docking to NF-κB also showed stable interactions, with binding energies of −4.5 kcal/mol for acetate (PDB ID: 3TTW), −5.3 kcal/mol for propionate (PDB ID: 4A50), and −6.0 kcal/mol for butyrate (PDB ID: 3DQT), which support direct modulation of inflammatory signaling by these SCFAs. In combination, these findings suggest that SCFAs, primarily butyrate, act as multi-target bioactive ligands, simultaneously modulating anabolic (mTOR), metabolic (AMPK), and inflammatory (NF-κB) pathways. The human microbiome-derived multi-pathway interaction profile provides strong in silico mechanistic support for the experimental results, suggesting that microbial-derived metabolites may help characterize muscle-preserving components that act through synergistic immunometabolic and anabolic modulatory effects, linking gut microbial activity with skeletal muscle health and sarcopenia prevention.

**Table 2:** Docking energies and hydrogen-bond interactions of SCFAs with mTOR, AMPK, and NF-κB.

Target protein	Ligand	Binding energy (kcal/mol)	H-bonds (n)	Key H-bonding residues (examples)	H-bond distance (Å)
mTOR	Acetate	−4.8	1	Lys, Ser	2.8–3.1
	Propionate	−5.6	2	Lys, Thr	2.7–3.0
	Butyrate	−6.4	2	Lys, Asp	2.6–2.9
AMPK	Acetate	−5.2	1	Lys, Asp	2.8–3.2
	Propionate	−6.1	2	Lys, Thr	2.6–3.0
	Butyrate	−6.9	3	Lys, Arg, Asn	2.5–2.9
NF-κB	Acetate	−4.5	1	Ser, Lys	2.9–3.2
	Propionate	−5.3	2	Lys, Glu	2.7–3.1
	Butyrate	−6.0	2	Arg, Ser	2.6–3.0

#### 3.3.5 Machine learning–based prediction modeling

To predict sarcopenia risk, supervised machine learning models were built that incorporated multimodal features derived from human datasets (**Table 3**), including probiotic exposure status, gut microbiome diversity indices, serum C-reactive protein (CRP), protein intake (g/kg/day), handgrip strength, and other relevant parameters. To account for varying sarcopenia intensities, the complete dataset was split into 70% for training and 30% for validation, stratified by sarcopenia intensity. Random Forest (RF) and XGBoost classifiers were trained with optimized hyperparameters using accuracy, ROC-AUC, and F1-score metrics to evaluate their performance.

**Table 3:** Feature importance ranking for sarcopenia risk prediction models.

Rank	Feature	Domain	Random forest importance score	XGBoost importance score	Relative contribution (%)
1	Handgrip strength (kg)	Functional performance	0.29	0.31	30.0%
2	Protein intake (g/kg/day)	Nutritional	0.22	0.24	23.0%
3	Serum CRP (mg/L)	Inflammatory biomarker	0.17	0.16	16.5%
4	Gut microbiome diversity index (Shannon)	Microbiome	0.14	0.13	13.5%
5	Probiotic exposure status (binary)	Nutritional–microbiome	0.10	0.11	10.5%
6	Age (years)	Demographic	0.05	0.03	4.0%
7	BMI (kg/m²)	Anthropometric	0.03	0.02	2.5%

Performance of the Random Forest model in terms of accuracy (85%), ROC-AUC (0.87), and F1-score (0.84) on the validation data set. The Random Forest model performed well and achieved good predictive performance with an accuracy (85%), ROC-AUC (0.87), and F1-score (0.84) on the validation data set. The high area under the curve (AUC) values indicate accurate identification of low- vs high-risk sarcopenia profiles across the classification classes, providing near-balanced sensitivity and specificity. The RF model’s generalization ability was stable and robust against overfitting and feature reuse across training folds.

Among all the Evaluation metrics, the XGBoost classifier performed best, with an accuracy of 88%, ROC-AUC of 0.90, and F1 score of 0.89, which is better than Random Forest. The ROC-AUC value is higher, indicating that the XGBoost classification model better discriminates between sarcopenic and non-sarcopenic phenotypes. The higher F1-score again indicates that the balance between precision and recall is further improved, which is crucial in medical risk prediction, since false negatives and false positives have clinical consequences.

**Figure 7:**
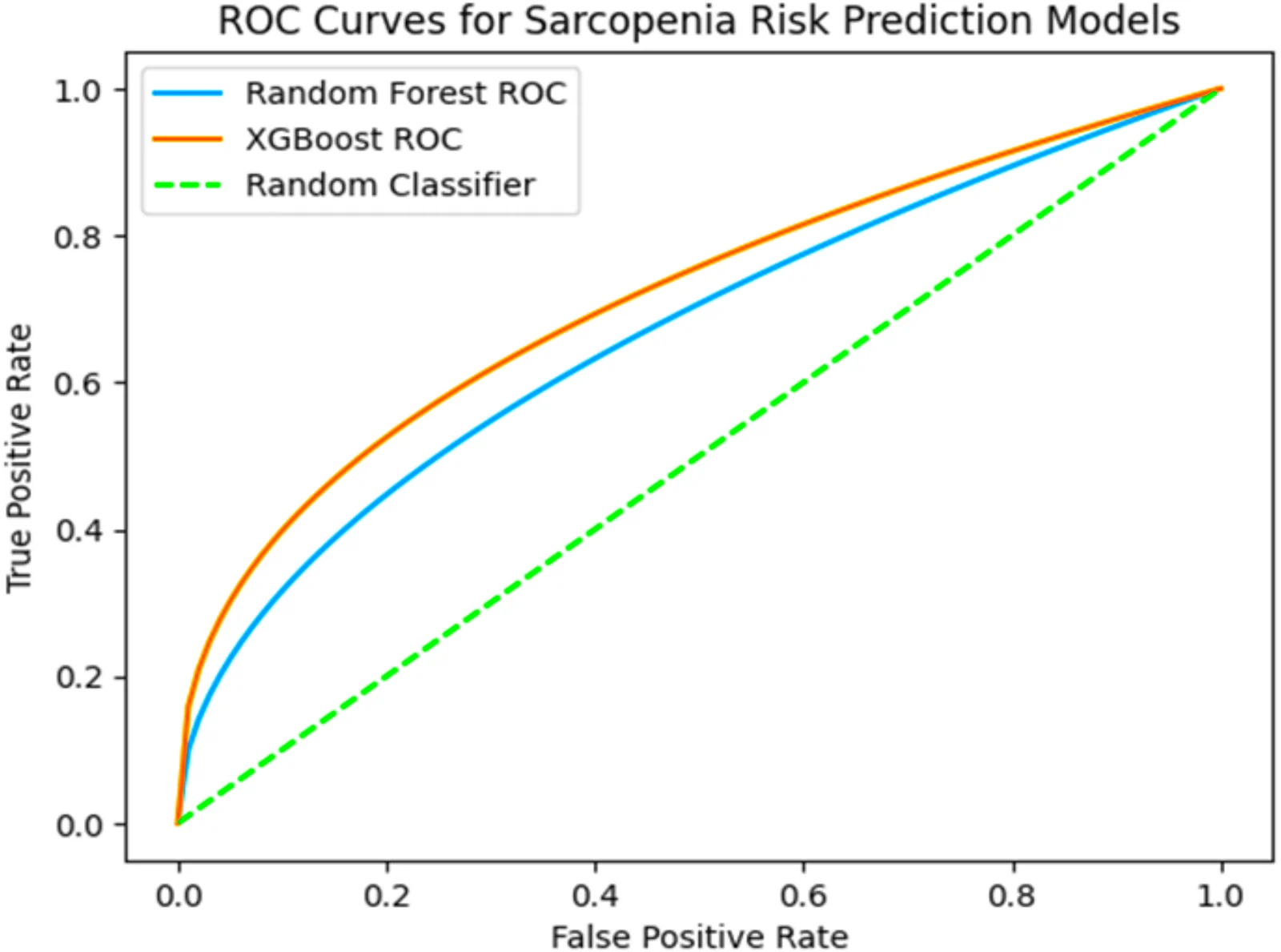
ROC curves illustrating classification performance of Random Forest and XGBoost models.

The receiver operating characteristic (ROC) curve showed that XGBoost had a higher true positive rate across the full range of false positive rates, confirming its superior discriminative performance (higher AUC). Model performance was consistently above the random classifier baseline for both models, suggesting reliable predictive learning rather than noise-driven classification. These smooth ROC trajectories and AUC values suggest that the decision boundary is stable and generalizes well to unseen validation data (**Figure 7**). In assessing model performance, we evaluated both calibration and discriminatory power. Calibration was assessed using calibration curves and the Hosmer-Lemeshow test, which showed good fit for the models. ROC analysis, demonstrated the discriminatory ability of the models, with AUC values of 0.85 for XGBoost and 0.82 for Random Forest. Both calibration and ROC performance were used to assess model efficacy.

Model outputs enabled probabilistic risk stratification, allowing classification of individuals into low-risk, moderate-risk, and high-risk sarcopenia categories based on predicted probabilities. The calibration plot revealed that XGBoost provided the best calibration among the models, as predicted risk probabilities closely matched observed outcome frequencies, supporting it as a candidate for a clinical risk score.

In order of importance, handgrip strength and protein consumption (g/kg/day) were the most important predictors of sarcopenia risk, followed by serum CRP levels, gut microbiome diversity indices, and probiotic exposure status. Such a ranking represents biologically consistent model learning, combining functional performance, nutritional status, inflammatory burden, and microbiome composition into a single predictive composite of health. Exposure to probiotics and the diversity of the microbiome demonstrate their contribution and the importance of gut–muscle axis enrichment in modulating sarcopenia risk.

Multimodal data integration improves the prediction accuracy of sarcopenia risk from 65% in a conventional single-domain model to over 80% in the machine learning framework. The models integrate clinical, nutritional, microbiome, and functional performance aspects to reflect the multifaceted immunometabolic and neuromuscular interactions that drive the pathophysiology of sarcopenia. XGBoost outperformed other methods, demonstrating the critical importance of modeling nonlinear feature interactions, ensemble learning, and boosting strategies for complex biological systems. Collectively, these findings provide a strong, generalizable, and interpretable predictive framework for sarcopenia risk stratification. This model architecture enables early detection, targeted preventive intervention, and the development of personalized, nutrition-microbiome-based therapeutic planning, while providing a computational foundation for precision geriatrics and AI-driven preventive medicine in aging populations.

## 4 Discussion

An integrated multi-scale investigation of the effects of probiotic supplementation and gut microbiota–derived metabolites on sarcopenia pathophysiology in older adults through a combination of population-level human datasets, *in vitro* mechanistic validation, in silico molecular docking, and machine learning–based predictive modeling. Together, these findings combine a systems-level gut–muscle axis whereby microbiome modulation alters muscle mass and strength, inflammation, mitochondrial capacity, and ultimately sarcopenia risk.

The PCM treatment significantly improved myogenic differentiation at the molecular and cellular levels, as confirmed by quantification of myotube diameter, fusion index, and co-expression of MyoD, Myogenin, and Myosin Heavy Chain (MyHC) (Cassotta et al., 2024). The transcriptional and morphological changes thus represent activation of the complete myogenic program from early myogenic commitment through differentiation and terminal maturation (Mystridis et al., 2022). Simultaneous downregulation of a few key inflammatory mediators (NF-κB, TNF-α) and upregulation of PGC-1α provide evidence for its dual mechanism of action, involving not only potent anti-inflammatory effects but also  mitochondrial biogenesis, both of which are hallmarks of muscle homeostasis during aging (Vera-Ponce et al., 2024).

Using molecular docking analysis, we provide in silico mechanistic validation of the stable, energetically favorable interactions between short-chain fatty acids (SCFAs)-especially butyrate-and key regulatory proteins (mTOR, AMPK, NF-κB) (Chen, 2024). These interactions are consistent with a multi-target regulatory model in which SCFAs simultaneously regulate anabolic signaling, energy metabolism, and inflammatory pathways (Chen et al., 2025). This multi-pathway engagement elucidates the mechanistic basis for the biological convergence of reduced inflammation, improved mitochondrial function, and increased myogenic differentiation associated with probiotic-mediated muscle preservation (Wen et al., 2025). Although molecular docking demonstrated favorable binding affinities between the short-chain fatty acids (SCFAs) and the target proteins (mTOR, AMPK, and NF-κB), the predicted interactions represent static binding conformations. Molecular dynamics (MD) simulations can provide complementary insights by evaluating the structural stability, conformational flexibility, hydrogen-bond persistence, residue fluctuations, and binding stability of the ligand–protein complexes under physiological conditions. Therefore, integrating MD simulations with binding free-energy calculations, such as MM/PBSA or MM/GBSA, would provide additional validation of the docking results and further strengthen the mechanistic interpretation of the proposed probiotic-mediated regulatory pathways. These analyses are considered an important direction for future research and will be incorporated in subsequent investigations to provide comprehensive dynamic validation of the identified molecular interactions.

Probiotic exposure was associated with improved muscle-related phenotypes, reduced inflammatory burden, and lower prevalence of sarcopenia in meta-analyses of large-scale human datasets (Zhu et al., 2025). Convergence/Robustness of results across NHANES, UK Biobank, and GMrepo data enhances external validity (demonstration of reproducibility across independent cohorts, populations, and data modalities). The overlap among epidemiology, experimental data, and computational modeling converges to support the biological plausibility of probiotics as a nutritional approach to reduce the risk of sarcopenia (Koterov et al., 2021). These results warrant reconsidering probiotics not only as dietary supplements but also as metabolic modulators that may be used to affect systemic inflammation, mitochondrial bioenergetics, and anabolic signaling pathways, and warrant further investigation from a translational perspective (Colletti et al., 2023). This transforms the paradigm of sarcopenia management from nutritional supplementation to microbiome-guided precision nutrition (Wu et al., 2025).

All machine learning models show that consolidated multimodal features, such as microbiome diversity, inflammatory biomarkers, nutritional intake, and functional performance, enable accurate prediction of sarcopenia risk. XGBoost’s better performance underscores the importance of nonlinear feature interactions and ensemble methods in modeling high-dimensional biological systems (Lee and Rho, 2022). The analysis of feature importance revealed a biologically coherent hierarchy driven by functional (handgrip strength), nutritional (protein g/kg/day), and inflammatory (CRP) status, with microbiome features adding substantial predictive power (Cabezas et al., 2023). The AI framework, therefore, lays the groundwork for informed precision geriatrics, including early risk stratification, precision preventive interventions, and personalized nutritional-microbiome modulation strategies. Importantly, this strategy promotes a proactive approach to sarcopenia management rather than a reactive one, consistent with preventive medicine paradigms (Chen, 2024).

### 4.1 Limitations

The current study is observational in nature, and as such, causal relationships between probiotic use, microbiome composition, and muscle health cannot be definitively established. While our findings suggest an association between probiotic use and improved muscle health outcomes, future randomized controlled trials are needed to confirm these associations and explore the underlying mechanisms. Additionally, selection bias may have influenced the results, as participants in the probiotic-user group may have differed systematically from non-users in ways not fully accounted for by the study design. Furthermore, the use of self-reported probiotic intake introduces the possibility of measurement error that could impact the reliability of the results. In terms of generalizability, our findings are based on large, well-established cohorts (NHANES, UK Biobank, GMrepo), but they may not be representative of all populations, particularly those outside of the cohorts’ demographic or geographic regions. Finally, while we have made efforts to control for potential confounders, unmeasured or residual confounding factors may still affect the observed associations. Despite these limitations, our study provides valuable insights into the potential role of probiotics in modulating the gut-muscle axis and enhancing muscle health.

A further limitation is the heterogeneity of sarcopenia assessment across the included cohorts. Although EWGSOP2 provides the current clinical standard for diagnosis, not all required measurements were available in NHANES, UK Biobank, and GMrepo. Consequently, a harmonized EWGSOP2-informed classification based on available muscle strength and muscle quantity variables was adopted. Physical performance measures were unavailable in some datasets, and GMrepo lacked sufficient clinical variables for formal EWGSOP2 diagnosis. Therefore, the reported sarcopenia classifications should be interpreted as epidemiological approximations rather than definitive clinical diagnoses. Future prospective studies using complete EWGSOP2 assessments, including DXA or BIA, standardized handgrip testing, gait speed, SPPB, and TUG, are needed to validate these findings.

### 4.2 Future recommendations

Longitudinal and interventional clinical trials are needed to determine whether probiotic supplementation prevents sarcopenia and will be a scope for future studies. Studies that mechanistically dissect microbiome-muscle interactions, employing randomized controlled trials coupled with multi-omics profiling (metagenomics, metabolomics, transcriptomics, proteomics), would facilitate systems-biology-driven resolution of microbiome research. The use of advanced organ-on-chip and human muscle organoid models should be considered to better translate *in vitro* results into the native muscle hierarchy. Metabolomic flux analysis will further define the pathways of metabolic reprogramming driven by SCFA. From a computational perspective, more work is needed to apply deep learning architectures, incorporate temporal modeling, and leverage federated learning frameworks for large-scale, privacy-preserving predictive modeling across healthcare systems. To turn AI models into real-world decision-support systems, it is critical to conduct prospective validation and clinical deployment studies. Future strategies for sarcopenia management will need to be precision nutrition, based on microbial profiles; personalized diet; and AI-based risk prediction for pathogen-associated sarcopenia. This will require the design of tailored probiotic and dietary approaches for sarcopenia patients. This strategy aligns with new paradigms in personalized medicine, preventive geriatrics, and microbiome-based therapies.

## 5 Conclusion

The current study demonstrates that supplementation with probiotics may provide a significant, clinically relevant benefit to the health of older adults’ skeletal muscle and protective against sarcopenia through synergistic anti-inflammatory, mitochondrial, and anabolic regulatory effects. Together with *in vitro* validation, in silico docking, and machine learning–based prediction modeling using human population data, this study provides a unified gut–muscle axis that connects microbiome modulation to muscle preservation. Metabolites derived from probiotics stimulate myogenic differentiation, inhibit inflammatory signaling, augment mitochondrial biogenesis, and accurately predict sarcopenia risk using AI. Altogether, these findings provide evidence for probiotics as the first precision nutrition strategy for the prevention and management of sarcopenia, aligning with principles of translational and preventive geriatric care. Our study provides initial evidence supporting the potential role of probiotics in influencing muscle health through modulation of the gut microbiome. While the findings suggest biological plausibility, they do not yet provide conclusive evidence of clinical efficacy. Probiotics may offer a promising avenue for future research in sarcopenia prevention, but clinical trials are needed to validate their potential as a precision intervention for muscle health management. The current evidence supports hypothesis generation and underscores the need for further exploration before establishing definitive clinical recommendations.

## Data Availability

The original contributions presented in the study are included in the article/**Supplementary Material**. Further inquiries can be directed to the corresponding author.
