# Supplementary material for: Probiotic supplementation as a nutritional strategy for the prevention and management of sarcopenia in older adults
Source: Front Cell Infect Microbiol. 2026 Aug 13;16:1801885. doi: 10.3389/fcimb.2026.1801885 (PMC13518184; doi:10.3389/fcimb.2026.1801885)
Supplement: Supplementary file 1 [file Table1.docx]

**Table S1: Composition of Probiotic-Conditioned Media (PCM)**

| **Component** | **Details** |
| --- | --- |
| **Probiotic Strains Used** | *Lactobacillus rhamnosus, Lactobacillus plantarum, Bifidobacterium longum* |
| **Culture Medium** | de Man, Rogosa, and Sharpe (MRS) broth |
| **Culture Conditions** | Anaerobic incubation at 37°C for 24 hours |
| **Probiotic Concentration** | 1×10⁹ CFU/mL |
| **Centrifugation** | 5,000 rpm for 10 minutes to remove bacterial cells |
| **Sterile Filtration** | 0.22 µm filter |
| **Storage Conditions** | Stored at -20°C until use |

**Table S2: Baseline Characteristics of NHANES Cohort**

| **Variable** | **Probiotic Users (n = 1,200)** | **Non-Users (n = 2,300)** | **p-value** |
| --- | --- | --- | --- |
| **Age (years)** | 69.3 ± 7.5 | 70.1 ± 7.8 | 0.025 |
| **Sex (Female)** | 52% | 50% | 0.45 |
| **BMI (kg/m²)** | 28.1 ± 4.2 | 29.0 ± 5.0 | 0.004 |
| **Protein Intake (g/kg/day)** | 1.1 ± 0.3 | 0.95 ± 0.28 | <0.01 |
| **Handgrip Strength (kg)** | 27.5 ± 4.8 | 25.1 ± 5.2 | <0.001 |
| **Fat-Free Mass (kg)** | 57.4 ± 10.6 | 56.2 ± 11.0 | 0.03 |
| **C-Reactive Protein (mg/L)** | 3.2 ± 4.5 | 3.9 ± 5.2 | 0.05 |

**Table S3: Baseline Characteristics of UK Biobank Cohort**

| **Variable** | **Probiotic Users (n = 1,500)** | **Non-Users (n = 3,500)** | **p-value** |
| --- | --- | --- | --- |
| **Age (years)** | 66.5 ± 6.8 | 67.2 ± 6.9 | 0.03 |
| **Sex (Female)** | 54% | 52% | 0.35 |
| **BMI (kg/m²)** | 27.4 ± 4.8 | 28.1 ± 5.2 | 0.01 |
| **Protein Intake (g/kg/day)** | 1.2 ± 0.3 | 1.1 ± 0.3 | 0.02 |
| **Handgrip Strength (kg)** | 27.2 ± 4.8 | 25.1 ± 5.2 | <0.001 |
| **Fat-Free Mass (kg)** | 57.4 ± 10.6 | 56.2 ± 11.0 | 0.04 |
| **C-Reactive Protein (mg/L)** | 3.1 ± 4.4 | 3.7 ± 5.1 | 0.06 |

**Table S4: Baseline Characteristics of GMrepo Cohort**

| **Variable** | **Probiotic-associated Microbiome (n = 500)** | **Low-probiotic Microbiome (n = 500)** | **p-value** |
| --- | --- | --- | --- |
| **Age (years)** | 68.4 ± 7.1 | 69.0 ± 6.9 | 0.12 |
| **Sex (Female)** | 51% | 53% | 0.40 |
| **BMI (kg/m²)** | 28.0 ± 4.1 | 28.3 ± 4.5 | 0.35 |
| **Protein Intake (g/kg/day)** | 1.1 ± 0.3 | 1.0 ± 0.3 | 0.05 |
| **Handgrip Strength (kg)** | 28.0 ± 4.7 | 25.5 ± 5.1 | <0.001 |
| **Fat-Free Mass (kg)** | 57.5 ± 9.8 | 56.1 ± 9.5 | 0.02 |
| **C-Reactive Protein (mg/L)** | 3.1 ± 4.6 | 3.8 ± 5.2 | 0.08 |
