# Supplementary material for: Probiotic supplementation as a nutritional strategy for the prevention and management of sarcopenia in older adults
Source: Front Cell Infect Microbiol. 2026 Aug 13;16:1801885. doi: 10.3389/fcimb.2026.1801885 (PMC13518184; doi:10.3389/fcimb.2026.1801885)
Supplement: Supplementary file 2 [file Table2.docx]

**Mathematical Harmonization of NHANES, UK Biobank, and GMrepo Data**

The NHANES, UK Biobank, and GMrepo datasets were collected from different sources and contain heterogeneous measurement scales, variable definitions, and data modalities. Therefore, a mathematical harmonization procedure was performed before machine learning model development to construct a unified feature representation and ensure compatibility across datasets.

Let the three datasets be represented as

[
D^{(1)}={X_{NHANES},Y_{NHANES}},
]

[
D^{(2)}={X_{UKB},Y_{UKB}},
]

[
D^{(3)}={X_{GMrepo},Y_{GMrepo}},
]

where (X) denotes the predictor variables and (Y) represents the sarcopenia outcome labels.

The common feature space was defined as

[
F={\text{Age},\ \text{Sex},\ \text{BMI},\ \text{Protein Intake},\ \text{Handgrip Strength},\ \text{CRP},\ \text{Microbiome Diversity},\ \text{Probiotic Exposure},\ \text{Muscle Index}}.
]

Each dataset was projected into the common feature space using the harmonization function

[
H_i=f(D_i), \qquad i=1,2,3
]

where (f(\cdot)) denotes the feature harmonization operator.

Missing values in continuous variables were estimated using Multiple Imputation by Chained Equations (MICE), expressed as

[
\hat{x}*{ij}=E(x*{ij}\mid X_{-j}),
]

where (X_{-j}) denotes the remaining observed variables used to estimate the missing value.

Continuous variables were standardized using z-score normalization,

[
z_{ij}=\frac{x_{ij}-\mu_j}{\sigma_j},
]

where (x_{ij}) is the original value of feature (j) for sample (i), while (\mu_j) and (\sigma_j) represent the mean and standard deviation of feature (j), respectively.

To eliminate measurement inconsistencies among cohorts, variables measured in different units (e.g., body composition and inflammatory biomarkers) were converted into common SI units prior to normalization.

Categorical variables, including sex and probiotic exposure, were transformed using binary encoding,

[
e(x)=
\begin{cases}
1, & \text{if probiotic user},\
0, & \text{otherwise}.
\end{cases}
]

Since microbiome sequencing data are compositional, microbial abundance was normalized as relative abundance using

[
A_i=\frac{c_i}{\sum_{k=1}^{n} c_k},
]

where (c_i) denotes the sequencing count of microbial taxon (i).

After preprocessing, the harmonized feature matrices from all three datasets were concatenated to generate a unified multimodal feature matrix,

[
X=
\begin{bmatrix}
H_1\
H_2\
H_3
\end{bmatrix},
]

with the corresponding outcome vector

[
Y=
\begin{bmatrix}
Y_1\
Y_2\
Y_3
\end{bmatrix}.
]

The integrated dataset was subsequently used for Recursive Feature Elimination (RFE), Synthetic Minority Over-sampling Technique (SMOTE), and the development of Random Forest and XGBoost classifiers. This mathematical harmonization minimized inter-dataset variability while preserving biologically meaningful information, thereby improving the robustness, reproducibility, and generalizability of the machine learning models for sarcopenia risk prediction.
